# A Novel Method to Predict Drug-Target Interactions Based on Large-Scale Graph Representation Learning

**DOI:** 10.3390/cancers13092111

**Published:** 2021-04-27

**Authors:** Bo-Wei Zhao, Zhu-Hong You, Lun Hu, Zhen-Hao Guo, Lei Wang, Zhan-Heng Chen, Leon Wong

**Affiliations:** 1 The Xinjiang Technical Institute of Physics & Chemistry, Chinese Academy of Sciences, Urumqi 830011, China; zhaobowei19@mails.ucas.ac.cn (B.-W.Z.); hulun@ms.xjb.ac.cn (L.H.); guozhenhao17@mails.ucas.ac.cn (Z.-H.G.); leiwang@ms.xjb.ac.cn (L.W.); huangliguang18@mails.ucas.ac.cn (L.W.); 2 University of Chinese Academy of Sciences, Beijing 100049, China; 3 Xinjiang Laboratory of Minority Speech and Language Information Processing, Urumqi 830011, China; 4 College of Computer Science and Software Engineering, Shenzhen University, Shenzhen 518060, China; chenzhanheng17@mails.ucas.ac.cn

**Keywords:** drug discovery, drug-target interactions, large-scale graph representation learning, computational method

## Abstract

**Simple Summary:**

The traditional process of drug development is lengthy, time-consuming, and costly, whereas very few drugs ever make it to the clinic. The use of computational methods to detect drug side effects greatly reduces the deficiencies in drug clinical trials. Prediction of drug-target interactions is a key step in drug discovery and repositioning. In this article, we proposed a novel method for the prediction of drug-target interactions based on large-scale graph representation learning. This method can be helpful to researchers in clinical trials and drug research and development.

**Abstract:**

Identification of drug-target interactions (DTIs) is a significant step in the drug discovery or repositioning process. Compared with the time-consuming and labor-intensive in vivo experimental methods, the computational models can provide high-quality DTI candidates in an instant. In this study, we propose a novel method called LGDTI to predict DTIs based on large-scale graph representation learning. LGDTI can capture the local and global structural information of the graph. Specifically, the first-order neighbor information of nodes can be aggregated by the graph convolutional network (GCN); on the other hand, the high-order neighbor information of nodes can be learned by the graph embedding method called DeepWalk. Finally, the two kinds of feature are fed into the random forest classifier to train and predict potential DTIs. The results show that our method obtained area under the receiver operating characteristic curve (AUROC) of 0.9455 and area under the precision-recall curve (AUPR) of 0.9491 under 5-fold cross-validation. Moreover, we compare the presented method with some existing state-of-the-art methods. These results imply that LGDTI can efficiently and robustly capture undiscovered DTIs. Moreover, the proposed model is expected to bring new inspiration and provide novel perspectives to relevant researchers.

## 1. Introduction

Drug repositioning is the process of exploring the new effects of existing drugs except for the original indications for medical treatment. It is a direction with great opportunities and challenges. In addition, it has the advantages of low-cost, short-time and low-risk [1,2]. The drug-target interactions (DTIs) play an important role in drug discovery and drug repositioning. Accurate prediction of DTIs can improve the accuracy of drug clinical trials, thus greatly reducing the risks of experiments. For a long time, the accumulation of a large number of biological experimental data and related literature makes the biological database richer and richer, which provides a favorable condition for the use of computational methods.

Traditional computing methods are mainly divided into two categories: ligand-based methods and structure-based methods. However, structure-based approaches are limited when the 3D structures of the target protein are absent, and ligand-based approaches have low accuracy when there are only a few binding ligands for the target protein [3,4,5,6,7]. In recent years, the widespread recognition of data-driven methods has made machine learning algorithms widely used in biomolecular correlation prediction [8,9,10,11]. There are mainly four related methods of in-silico methods: machine learning-based methods, network-based methods, matrix factor-based methods, and deep learning-based methods [12,13,14]. For example, Ding et al. [15] used substructure fingerprints, physical and chemical properties of organisms, and DTIs as feature extraction methods and input features, and further used SVM for classification. Chen et al. [16] employed gradient boosting decision tree (GBDT) to predict drug-target interactions based on three properties, including IDs of the drug and target, the descriptor of drug and target, DTIs. Luo et al. [17] constructed a heterogeneous network to predict the potential DTIs by integrating the information of multiple drugs. Chen et al. [18] and *Ji* et al. [19] proposed a multi-molecular network model based on network embedding to predict novel DTIs. Liu et al. [20] proposed a model called NRLMF, which calculates the score of DTIs through logical matrix decomposition, where the properties of the drug and target are expressed in terms of their specificity. Zheng et al. [21] proposed to map the drug and target into a low-rank matrix and to establish the weighted similarity matrix, and solve the problem by using the small square algorithm. Wen et al. [22] used unsupervised learning to extract representations from the original input descriptors to predict DTIs.

Recently, the extensive application of non-Euclidean structured data in graph neural networks has led to various graph-based algorithms [23,24,25,26,27,28,29,30], such as graph convolution networks (GCN), graph attention networks (GAT), graph autoencoders (GAE), graph generative networks, graph spatial-temporal networks, etc. Based on the analysis of biological data, it is found that the biological data network has a good preference for the graph neural network. Gao et al. [31] used long short-term memory (LSTM) and graph convolutional networks (GCN) to represent protein and drug structures, to predict DTIs. Previous work has shown the preferable performance of graph neural network for DTIs [27,32], however, a single understanding of the data relationship between DTIs cannot mine out the hidden information of the graph data well. Therefore, it is necessary to explore the depth information of the drug and target protein through the graph neural network.

In the actual graph, the relationship between two nodes is complex, and the features of each node are usually composed of a variety of attributes. It is necessary to clearly understand the relationship between nodes. Therefore, the extraction of node features should be multi-angle and multi-dimensional. To solve these challenges, we propose a novel method to predict DTIs based on large-scale graph representation learning (LGDTI). Unlike previous graph-based neural network-based approaches, LGDTI aims to gain an in-depth understanding of known drugs and targets association networks through different graph-based representation learning methods. To extract hidden graph features of drugs and targets in a complex biological network, two types of graph representation learning were used to excavate them.

## 2. Materials and Methods

### 2.1. Datasets

In this article, the multi-graph data were collected from DrugBank5.0 [33]. DrugBank5.0 is an open, free, comprehensive database, including drug molecular structures, mechanisms, and drug-target interactions that are constantly being updated. We downloaded 11,396 known DTIs from Drugbank5.0, including 984 drugs and 635 proteins; 11,396 known DTIs are conducted as the benchmark dataset, and in training as the positive sample.

### 2.2. Drug Attribute Representation

**Figure 1:**
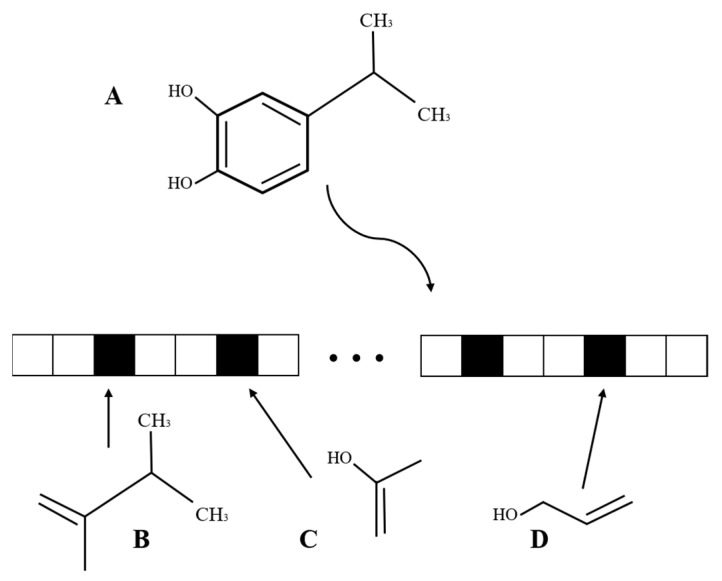
A schematic diagram of the drug molecular structure is constructed as bit vectors. A is the structure of a drug molecule, and B, C, and D are all substructures of the drug molecule, corresponding to the converted bit (represented by the small black box), respectively.

The molecular structure of the drug was extracted from the DrugBank database. The molecular structure is complex and difficult to use directly. To facilitate the calculation of drug molecular structure, it was necessary to vectorize its molecular structure [34]. The molecular fingerprint [35] is an abstract representation of a molecule, which encodes a molecule as a series of bit vectors, in which each bit on the molecular fingerprint corresponds to a molecular fragment, as shown in Figure 1. For the drug data, RDKit [36] was selected to calculate the Morgan fingerprint of the drug molecule.

### 2.3. Protein Attribute Representation

Protein sequence information was extracted from the STRING database [37]. Proteins are important biological macromolecules. All proteins are polymers formed by the linkage of 20 different amino acids, including (Ala, Val, Leu, Ile, Met, Phe, Trp, Pro), (Gly, Ser, Thr, Cys, Asn, Gln, Tyr), (Arg, Lys, His), and (Asp, Glu). Subsequently, the k-mer method is used [38], and k is set to 3, which translates each protein sequence into a 64-dimensional (4 * 4 * 4) feature vector by calculating the occurrence frequency of each sub-sequence in the entire protein sequence.

### 2.4. Graph Convolutional Network for Drug-Target Interactions (DTIs)

A graph convolutional network (GCN) [39] is a semi-supervised approach that turns topological associations into topological diagrams. In the algorithm, the input of GCN is the structure of the graph and the characteristics of each node, and the output includes the results at the node level, the results at the graph level, and the pooling information at the node level. Consequently, it is widely used in non-Euclidean spaces.

Let us assume that we have a bipartite graph G= with $V=\left[v_{1},\cdots ,v_{n},\cdots ,v_{m+n}\right]$ representing n drugs and m proteins, $E=\left[e_{ij}\right]$ representing the relationship of drug i and protein j. If $e_{ij}=1$, $v_{i}$ and $v_{j}$ has a connection. Furthermore, in the graph the attributes of all nodes $X=\left[\begin{array}{c} X_{d}\\ X_{p} \end{array}\right]$, the attributes of the drug $X_{d}={\left[x_{1}^{d},\cdots ,x_{n}^{d}\right]}^{T}$ and the attributes of the protein $X_{p}={\left[x_{1}^{p},\cdots ,x_{m}^{p}\right]}^{T}$.

In this work, we define the function f(X,A) using the spatial method of GCN, where X is the feature set of each node, and A is the adjacency matrix. Therefore, the network communication rules of GCN are as follows:

(1) $$f{\left(X,A\right)}^{l+1}=\sigma \left({\tilde{D}}^{-\frac{1}{2}}\tilde{A}{\tilde{D}}^{-\frac{1}{2}}X^{l}W^{l}\right),$$

in which, $\tilde{A}=A+I_{n+m}$ is the adjacency matrix added to the self-loop, $\tilde{D}$ is represented as the degree matrix of $\tilde{A}$. W is the weight of the randomly initialized the network. σ represents the activation function of each layer of the neural network, here σ is ReLU(·).

Although GCN has a natural preference for graph data, for DTIs data, we finally determined l=1 and W is 64 * 64 after analysis and experiment. Then, in the initial training, we found that the algorithm had the problem of over-smoothing. To solve this challenge, we adjusted the defect of the original algorithm for this data. Specifically, after each convolution, we added node features for training, the formula is as follows:

(2) $$f{\left(X,A\right)}^{l+1}=Τ\left(\sigma \left({\tilde{D}}^{-\frac{1}{2}}\tilde{A}{\tilde{D}}^{-\frac{1}{2}}X^{l}W^{l}\right),X\right),$$

we adopted this adjusted graph convolution definition in this work.

### 2.5. Graph Embedding—DeepWalk for DTIs

DeepWalk [40] is a method to learn the potential representation of nodes in a graph and is a widely used algorithm in graph embedding. The main idea of the algorithm is divided into two parts. The first part is to sample the graph based on the random walk and map the node adjacency structure into sequence structure. The second part is to train the Skip-gram model by using the sequences obtained from sampling so that the expression of learning can capture the connectivity between nodes. Let us assume that we have a bipartite graph G=(V,E). V is the set of nodes in the graph, and E is the edge of nodes. Each calculation starts from a given starting point, and then carries out a random walk through the sampled neighbor nodes, repeating the operation until the length of the sampled sequence is equal to the given maximum length, as shown in Algorithm 1.

(3) $$S_{i}=(v_{i}|\left(v_{1},v_{2},v_{3},\cdots ,v_{i-1}\right)),$$

where, $S_{i}$ is the random walk collection sequence, and $v_{i}$ is the random node.

Therefore, in the second part of the algorithm, S is computed by the Skip-gram model. Specifically, a two-layer neural network model is established. The input is the node sequence matrix of $S^{n*m}$, and the weights in the neural network model are set as $W_{1}^{m*h}$ and $W_{2}^{h*m}$ respectively. Secondly, through backpropagation, the weight parameters are updated to obtain the representation of the target node, as shown in Algorithm 2.

(4) $$S={\left[S_{1}^{m},S_{2}^{m},\cdots ,S_{n}^{m}\right]}^{T},$$

**Algorithm 1 DeepWalk** (G,w,d,γ,)**Input:** graph G(V,E)
  windows size w
  representation size d
  epoch γ
  step length t
**Output:** matrix of nodes representation $\psi \in {\mathbb{R}}^{\left|V\right|\times d}$
1: Initialization: ψ
2: Build a binary Tree T from V
3: **for**
i=0 to μ
**do**4:  $V^{\prime }$= **Shuffle**(V)5: **for each**
$v_{i}\in V^{\prime }$
**do**6:   $M_{v_{i}}=RandomWalk\left(G,v_{i},t\right)$
7:   $SkipGram\left(\psi ,M_{v_{i}},w\right)$
8: **end for**9: **end for**

**Algorithm 2 SkipGram** ($\psi ,M_{v_{i}},w$)1: **for each**
$v_{j}\in S_{v_{b}}$ do2: **for each**
$u_{k}\in S_{v_{i}}\left[j-w:j+w\right]$
**do**3:   $J\left(\psi \right)=-logPr(u_{k}|\psi \left(v_{j}\right))$
4:   $\psi =\psi -\alpha \times \frac{\partial J}{\partial \psi }$
5: **end for**6: **end for**

### 2.6. Construction of the Large-Scale Graph Representation Learning Network

Given a graph G(V,E) containing vertices V and edges E, where $e_{ij}$ is regard as a connection of $v_{i}$ and $v_{j}$. a graph is considered as an adjacency matrix or an incidence matrix [41]. For an adjacency matrix A, $A\in R^{N\times N}$, is defined as:

(5) $$A_{ij}=\{\begin{array}{l} 1\;\mathrm{if}\;\left(v_{i}{,v}_{j}\right)\subseteq E\\ 0\;\mathrm{else}\; \end{array},$$

Here, we used an undirected cycled graph, so $a_{ii}=1$. For an incidence matrix B, $B\in R^{N\times M}$, is defined as:

(6) $$B_{ij}=\{\begin{array}{l} 1{\;\mathrm{if}\;v}_{i}{\;\mathrm{and}\;v}_{j}\;\mathrm{are}\;\mathrm{connected}\\ 0\;\mathrm{else}\; \end{array},$$

The function of graph representation learning is to map data from complex graph space to multi-dimensional space. Its form is as follows:

(7) $$f:V\to X\in {ℜ}^{d},$$

where d≪|V|, $V=\left[v_{1},v_{2},v_{3},\cdots ,v_{n+m}\right]$ is the original set of spatial variables and $X=\left[x_{1},x_{2},x_{3},\cdots ,x_{d}\right]$ is the projected vector (or the embedded vector) that contains the structural information.

**Figure 2:**
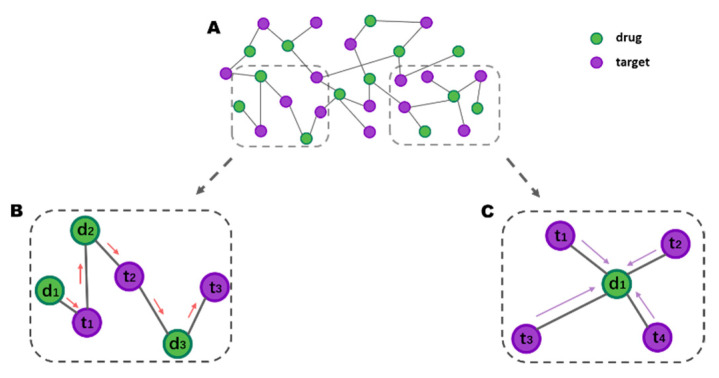
An example of large-scale graph representation learning. (**A**) The schematic diagram of the relationship between drugs and targets. (**B**) An example of the graph embedding in drug-target interactions (DTIs). (**C**) An example of the graph convolutional network.

The first-order information is generally used to describe the local similarity between pairs of vertices in a graph [42]. Specifically, if there is an edge between two vertices, the two vertices should be close to each other in the embedded space. If there is no edge connection between two vertices, the first-order proximity between them is 0. Such work usually uses the KL-divergence [43] to calculate the distance by minimizing:

(8) $$O_{1}=-\sum_{(i,j)\in E}^{}w_{ij}\log p_{i}\left(v_{i},v_{j}\right),$$

in which $p_{1}\left(v_{i},v_{j}\right)=\frac{1}{1+\exp(-v_{i}^{T}\cdot v_{j})}$, $v_{i}$ and $v_{j}$ are the low-dimensional vector representation of the node $v_{i}$ and $v_{j}$. $W_{ij}$ is the edge weight between node i and j. Although the methods based on the first-order neighbor of nodes are successful in graph embedding, they often fail to combine node substructure and node attributes for optimization. To address this challenge, the advantages of graph convolutional networks in vertex local feature extraction are utilized in Equation (1) to remedy this defect. An example of this algorithm is shown in Figure 2C.

The high-order information is learning the relationship between vertex $v_{i}$ and the other vertices separately [44,45]. Although there is no direct connection between the two vertices in the high-order information, learning that their representation vectors are close means that they should have similar or identical neighbors in the actual relational graph. For example, Figure 2B shows that drug $d_{1}$ has a second-order relationship with the target $t_{2}$, drug $d_{2}$ and drug $d_{1}$ have a shared target $t_{1}$, and target $t_{3}$ is a high-order potential candidate for drug $d_{1}$. Then, we abstract high-order information (or global structure information) for each node by the graph embedding method: DeepWalk.

Consequently, we constructed a large-scale graph representation learning network to learn the features of each node, as shown in Figure 2. In which Figure 2A is the drug-target interactions sub-network.

### 2.7. The Large-Scale Graph Representation Learning DTI (LGDTI) Model Framework

**Figure 3:**
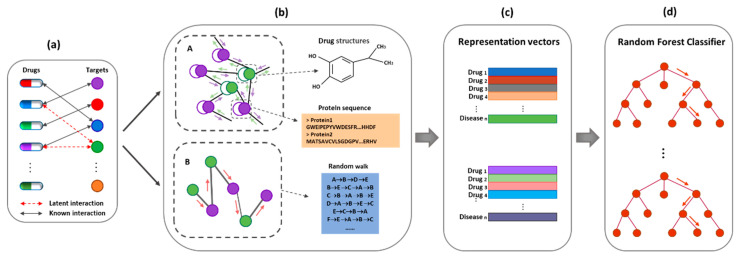
The flowchart of the proposed large-scale graph representation learning DTI (LGDTI). (**a**) A bipartite graph of DTIs. The solid black line is described as known DTIs, and the dashed red line is described as latent DTIs. (**b**) Part A constructed an adjacency graph containing a self-loop, in which green nodes are drugs and purple nodes are targets, and the information of *first-order* neighbors of each node is aggregated through graph convolutional network. Part B represented *high-order* information of each node in a bipartite graph by DeepWalk. (**c**) The two kinds of representation features are integrated. (**d**) Random forest classifier is trained and used for predicting new DTIs.

In this study, the proposed LGDTI model contains not only first-order but also high-order graph information. In the first-order graph information, the graph convolutional network is used to capture the first-order neighbor information of the nodes in the graph; in the high-order graph information, the graph embedding algorithm DeepWalk is used to capture the high-order neighbor information of the nodes in the graph. Through these two different methods, the local and global information of each node in the graph is captured by LGDTI. The first-order neighbor information contains the attributes of nodes, which are internal to the node; the high-order neighbor information contains the whole network information of the node, which is called the behavior information. In the end, the two kinds of representation features of nodes obtained from LGDTI are predicted by the random forest classifier. The framework of large-scale graph representation learning as shown in Figure 3. In short, we have three main contributions: (i) we propose to employ specific GCN to learn first-order neighbors’ information (or local structural information) of nodes. (ii) This article proposes to utilize a graph embedding algorithm to learn high-order neighbors’ information (or global structural information) of nodes. (iii) In conclusion, LGDTI can view the DTIs network from multiple perspectives, including three features in the whole feature extraction process: node attributes, node first-order information, and node high-order information. 

## 3. Results and Discussion

### 3.1. Performance Evaluation of LGDTI Using 5-Fold Cross-Validation

**Figure 4:**
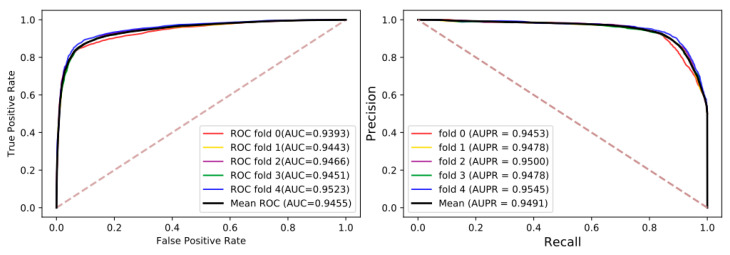
The receiver operating characteristic (ROC) and precision-recall (PR) curves under 5-fold cross-validation.

To accurately evaluate the stability and robustness of LGDTI, 5-fold cross-validation was adopted. In detail, the original data set was randomly divided into 5 subsets, among which 4 subsets were selected for each training, and the remaining subsets were used as the test set and repeated 5 times. Additionally, we used five evaluation indicators, including Acc. (Accuracy), MCC. (Matthews’s Correlation Coefficient), Sen. (Sensitivity), Spec. (Specificity), and Perc. (Precision). Moreover, for binary classification, the receiver operating characteristic (ROC) curve can reflect the capability of the model, while the AUC is the area under the ROC curve. The closer the ROC curve is to the upper left corner, the better the performance of the model. Similarly, the value of AUC is also high. The precision-recall (PR) curve contains precision and recall, with recall as the horizontal axis and precision as the vertical axis. On very skewed data sets, the PR curve can give us a comprehensive understanding of the performance of the model. The details of LGDTI under 5-fold cross-validation are shown in Table 1 and Figure 4. The results of each fold AUC, AUPR, and various evaluation criteria show that the proposed method has a better predictive ability. Studying it carefully, the results of each training are close to each other, which shows that the model has preferable stability and robustness.

### 3.2. Comparison LGDTI with the Different Machine Learning Algorithms

**Figure 5:**
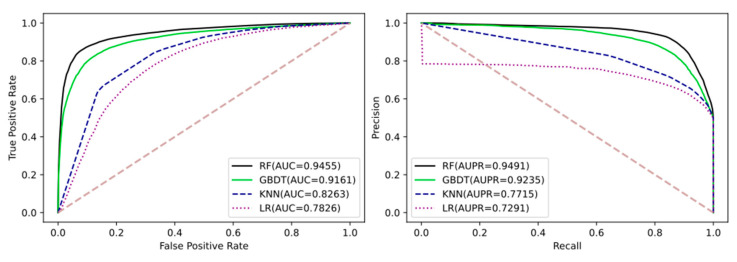
Comparison of the ROC and PR curves performed based on different machine learning classifier.

Different machine learning algorithms have different representations of features. By comparing different classification algorithms, including logistic regression (LR), K-nearest neighbor (KNN), gradient boosting decision tree (GBDT), and random forest classifier (RF), we can intuitively see the feature advantages of LGDTI. To make the comparison fairer and more objective, all classification algorithms choose the default parameters. The detailed evaluation results of 5-fold cross-validation are shown in Table 2 and Figure 5.

The results can be explained as follows: (i) for logistic regression, because of the depth and high complexity of input features, it may be difficult to form a linear classification surface, so it is impossible to fit features; (ii) for K-nearest Neighbor, in the characteristics of the sample studied in the early stage, the attributes of the neighboring nodes in the sample have been fused, which makes it impossible to accurately compare K-nearest neighbor; (iii) gradient boosting decision tree and random forest classifier are both ensemble classifiers, which can better solve the shortcomings of a single classifier, especially the random forest classifier, which can achieve preferable results on this dataset.

### 3.3. Comparison of the Different Feature with Attribute, GF and LGDTI

**Figure 6:**
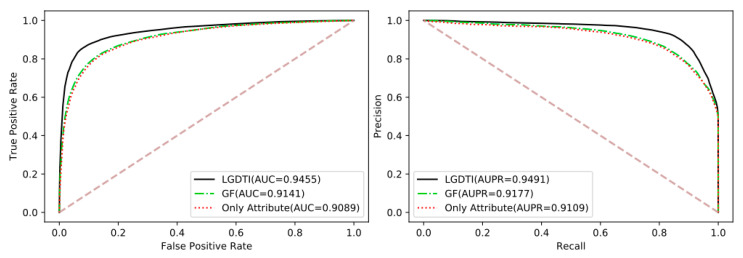
Comparison of the ROC and PR curves performed by random forest classifier based on different features.

In summary, LGDTI constructs a graph and combines the first-order and high-order information of the nodes in the graph to denote the characteristics of each node. The first-order graph information aggregates the direct neighbor information of nodes. In graph theory, two nodes have similarities if the structure is similar to the subgraph. The high-order graph information provides a preferable representation of each node’s indirect neighbor information. Therefore, we conducted experiments on the different features of nodes, in which random forest classifier was used, as shown in Table 3 and Figure 6. In Table 3, Attribute has exemplified the feature of drug molecular structure and protein sequence; only first-order graph information is represented as GF; LGDTI includes the first-order and high-order graph information. When only node self-attributes are the worst, while self-attributes of nodes can be enhanced through GCN. Therefore, only the combination of first-order graph information and high-order graph information can better explore the potential features of nodes.

### 3.4. Compared with Existing State-of-the-Art Prediction Methods

To evaluate the advantage of the proposed method, it is compared with other advanced methods. Although the method proposed by Chen et al. [18] and Ji et al. [19], considers the network information of nodes, it fully expressed the local information of nodes in the network. Then, LGDTI is relatively sufficient for information extraction of nodes, and its high AUROC, AUPR, and ACC are stronger than other methods, as shown in Table 4.

Compared with other methods, node attributes (LGDTI (Only Attribute)), node first-order information (LGDTI (GF)), and the LDGTI model are all better. Among them, in the case of only node attributes, the AUROC, AUPR, and ACC of our model are at least 0.031, 0.0281, and 0.0259 higher respectively. Meanwhile, LGDTI (GF) still has some advantages. Definitively, the AUROC, AUPR, and ACC of the LGDTI model are at least 0.0222, 0.019, and 0.0281 higher than that of Ji et al. methods (Attribute+Behavior), respectively. The first-order neighborhood information aggregation makes node attribute characteristics are enhanced. Furthermore, the integration of first-order information and high-order information of the node will make our method have better prediction ability.

### 3.5. Case Studies

To test the practical ability of our model, the drugs clozapine and risperidone were exploited to predict potential targets, respectively. Clozapine can be used to treat many types of schizophrenia, and it can directly inhibit the brain stem reticulum up-activation system and has a powerful sedative and hypnotic effect. Risperidone is a psychiatric drug used to treat schizophrenia. In particular, it has an improved effect on the positive and negative symptoms and their accompanying emotional symptoms. It may also reduce the emotional symptoms associated with schizophrenia. In this case study, all known associations in the benchmark dataset were trained by our method, and we sorted the predicted scores of the remaining candidate targets and selected the top 5 targets, as shown in Table 5. The experiment showed that there were 3 targets of the drugs clozapine and risperidone predicted by LGDTI, which could be proved in the SuperTarget database [46]. The remaining unproven targets may be candidates, hopefully, to be explored by medical researchers.

## 4. Conclusions

Although the accurate and efficient computational model could greatly accelerate the process of identification of DTIs, there is still a huge gap between academia and industry. In this study, we developed a novel method called LGDTI for predicting DTIs. Specifically, the nodes in LGDTI can be represented by 2 kinds of feature including first-order information learned by GCN and high-order information learned by DeepWalk from the graph. in which molecular fingerprint technology was used to extract the attribute of drugs, and the k-mer method was used to extract the attribute of targets. Then, the Random Forest classifier was applied to carry out the relationship prediction task. The presented method obtained the AUC of 0.9455 and the AUPR of 0.9491 under 5-fold cross-validation which is more competitive than several state-of-the-art methods. Moreover, our method can learn three kinds of information about the node, including the node’s attributes, local structure, and global structure. Specifically, LGDTI can integrate attribute information with structural information for learning. The experimental results show that LGDTI has a prominent predictive ability for DTIs. Nevertheless, due to the limitation of the benchmark dataset, the performance of LGDTI cannot be shown collectively in multiple data. Moreover, LGDTI may be greatly improved if two kinds of node information can be better integrated. Consequently, we hope that the proposed model could be utilized to guide drug development and other biological wet experiments.

## Figures and Tables

**Table 1:** Five-fold cross-validation results by random forest classifier.

Fold	Acc. (%)	MCC (%)	Sen. (%)	Spec. (%)	Prec. (%)	AUC (%)
0	88.36	77.11	83.25	93.46	92.72	93.93
1	88.60	77.54	83.90	93.29	92.59	94.43
2	88.22	76.89	82.85	93.60	92.83	94.66
3	88.40	77.22	83.16	93.64	92.90	94.51
4	89.61	79.52	85.18	94.04	93.46	95.23
Average	88.64 ± 0.56	77.66 ± 1.07	83.67 ± 0.93	93.61 ± 0.28	92.90 ± 0.33	94.55 ± 0.47

**Table 2:** Comparison of different machine learning classifier.

Classifier	Acc. (%)	MCC (%)	Sen. (%)	Spec. (%)	Prec. (%)	AUC (%)
LR	72.54 ± 1.23	45.23 ± 2.47	76.57 ± 1.29	68.51 ± 1.49	70.86 ± 1.21	78.26 ± 0.78
KNN	71.07 ± 1.15	46.90 ± 1.82	92.99 ± 0.68	49.15 ± 2.69	64.67 ± 1.09	82.63 ± 0.46
GBDT	84.98 ± 0.23	70.23 ± 0.41	80.54 ± 0.65	89.41 ± 0.26	88.38 ± 0.19	91.62 ± 0.38
RF	88.64 ± 0.56	77.66 ± 1.07	83.67 ± 0.93	93.61 ± 0.28	92.90 ± 0.33	94.55 ± 0.47

**Table 3:** Comparison of different feature using random forest classifier.

Feature	Acc. (%)	MCC (%)	Sen. (%)	Spec. (%)	Prec. (%)	AUC (%)
Attribute	83.86 ± 0.32	67.78 ± 0.65	81.62 ± 0.69	86.09 ± 0.56	85.44 ± 0.47	90.89 ± 0.38
GF	84.28 ± 0.46	68.76 ± 0.90	80.67 ± 0.89	87.90 ± 0.62	86.96 ± 0.56	91.41 ± 0.36
LGDTI	88.64 ± 0.56	77.66 ± 1.07	83.67 ± 0.93	93.61 ± 0.28	92.90 ± 0.33	94.55 ± 0.47

**Table 4:** Compared with existing state-of-the-art prediction methods.

Methods	Datasets	AUROC	AUPR	ACC
Ji et al. methods (Only Attribute)	DrugBank	0.8777	0.8828	0.8073
Chen et al. methods (Only Attribute)	DrugBank	0.8779	N/A	0.8127
LGDTI (Only Attribute)	DrugBank	0.9089	0.9109	0.8386
LGDTI (GF)	DrugBank	0.9141	0.9177	0.8428
Chen et al. methods (Only Behavior)	DrugBank	0.9206	N/A	0.8545
Ji et al. methods (Only Behavior)	DrugBank	0.9218	0.9286	0.8575
Ji et al. methods (Attribute+Behavior)	DrugBank	0.9233	0.9301	0.8583
LGDTI	DrugBank	0.9455	0.9491	0.8864

**Table 5:** New association prediction results for the top 5 targets with clozapine and risperidone.

Drug Name	Target Name	Confirmed
Clozapine	Alpha-1D adrenergic receptor	SuperTarget
	Cytochrome P450 3A5	SuperTarget
	UDP-glucuronosyltransferase 1A1	Unconfirmed
	Solute carrier family 22 member 3	Unconfirmed
	Sodium-dependent serotonin transporter	SuperTarget
Risperidone	Alpha-1D adrenergic receptor	SuperTarget
	Solute carrier family 22 member 8	Unconfirmed
	Cytochrome P450 2C19	Unconfirmed
	Sodium-dependent serotonin transporter	SuperTarget
	Potassium voltage-gated channel subfamily H member 2	SuperTarget

## Data Availability

The data presented in this study are available on request from the corresponding author.
